# Factors Influencing Uptake of Breastfeeding: The Role of Early Promotion in the Maternity Hospital

**DOI:** 10.3390/ijerph18094783

**Published:** 2021-04-30

**Authors:** Rosalia Ragusa, Marina Marranzano, Valentina Lucia La Rosa, Gabriele Giorgianni, Elena Commodari, Rosalba Quattrocchi, Salvatore Cacciola, Vincenzo Guardabasso

**Affiliations:** 1 Health Technology Assessment Committee, University Hospital “G. Rodolico—San Marco”, 95123 Catania, Italy; ragusar@unict.it; 2 Department of Medical and Surgical Sciences and Advanced Technologies “G.F. Ingrassia”, University of Catania, 95123 Catania, Italy; marranz@unict.it (M.M.); giorgiangab@gmail.com (G.G.); 3 Department of Educational Sciences, University of Catania, 95124 Catania, Italy; e.commodari@unict.it; 4 Health Promotion Unit, University Hospital “G. Rodolico—San Marco”, 95123 Catania, Italy; quattrocchi@policlinico.unict.it; 5 Health Education Unit, Azienda Sanitaria Provinciale 3, 95124 Catania, Italy; scacciola59@gmail.com; 6 Research Promotion Office, University Hospital “G. Rodolico—San Marco”, 95123 Catania, Italy; guardabasso@policlinico.unict.it

**Keywords:** breastfeeding initiation, breastfeeding cessation, early postpartum, promotion of breastfeeding, nursing support

## Abstract

Background: This study aimed to explore the prevalence of breastfeeding at birth points in Sicily and the relevance of the factors influencing the adoption of exclusive breastfeeding during hospitalization linked to childbirth. Methods: A survey was conducted to monitor the prevalence of breastfeeding in seven out of nine facilities providing maternity services in the province of Catania (Sicily, Southern Italy) in the years 2016–2018. An online questionnaire was administered using an electronic tablet by the midwife to the mother after discharge. Results: Women who had a higher educational qualification breastfed in a greater proportion (59.6%; odds ratio OR 0.60 for abandoning breastfeeding). Having had a caesarean section moderately impaired breastfeeding uptake, with an almost double chance of declining exclusive breastfeeding (OR = 1.74). Starting breastfeeding within 1 h from delivery showed a significant facilitating effect (OR = 0.58). Rooming-in had a strong facilitating effect on exclusive breastfeeding. A breastfeeding advocacy program was shown to protect from abandoning breastfeeding. Conclusions: It is important to offer in all hospitals the possibility and support for breastfeeding in the first moments after childbirth to increase the number of those who will then continue with exclusive breastfeeding.

## 1. Introduction

In 1990 the WHO launched a campaign to promote breastfeeding [1], but the data show that those goals have been achieved in almost no country in Europe, starting with Italy [2]. According to WHO recommendations, only in Finland mothers do breastfeed their babies “exclusively” for six consecutive months [3,4]. Recently, during the World Breastfeeding Week 2020, WHO reiterated the need for increased financing for breastfeeding programs to improve the percentage of breastfeeding mothers [5].

The Italian Society of Paediatrics published a recommendation favoring breastfeeding, which would be contraindicated only for a suitable medical reason [6,7]. Even in the case of infectious diseases, there is never an absolute contraindication to breastfeeding [8,9]. Furthermore, breastfeeding has important benefits for maternal health, such as reducing the risk for some cancers (breast cancer, ovarian cancer, and endometrial cancer) [10,11,12,13,14].

Breastfeeding is healthy and right, but it is also tiring, complicated, and binding. Mothers may not always be willing or able to do it. Guilt feelings often appear in the non-nursing mother [15,16].

Although people in Italy are being definitely pro-breastfeeding [17], according to the Italian National Institute of Statistics (ISTAT) data [18], the average number of months of exclusive breastfeeding in the year 2013 was 4.1: the highest value was observed in the Autonomous Province of Trento (5.0), and the lowest in Sicily (3.5 months). This study’s objective was to carry out an observation of the prevalence of breastfeeding at birth points in Sicily and to explore the relevance of some factors influencing the adoption of exclusive breastfeeding during hospitalization linked to childbirth. This study was aimed at describing the present situation in a local setting, to be able to further promote and encourage breastfeeding in correspondence with locally relevant factors.

The mother’s characteristics, elements related to childbirth, and the different types of hospital structures that host the woman about to give birth were analyzed to evaluate the influence of these factors on exclusive breastfeeding.

While the psychological aspects and the degree of satisfaction of the woman with delivery experience are difficult to assess, with this study, we intend to verify whether external factors, such as attendance to appropriate courses, could be a factor that protects against the risk of abandoning exclusive breastfeeding.

We also intended to evaluate whether support during the first moments after childbirth can promote early attachment and breastfeeding uptake.

## 2. Materials and Methods

The Health Education Operating Unit of the local Health Agency in Catania, Italy had carried out a survey to monitor the prevalence of breastfeeding in seven out of nine facilities providing maternity service centers in the province of Catania (Sicily, Southern Italy) in years 2016–2018, as part of the preventive action of the National Prevention Plan [19].

An online questionnaire, created ad hoc (Google Forms, Google LLC, Mountain View, CA, USA), was used and administered using an electronic tablet by the midwife to the mother after discharge (computer-aided personal interview). The purpose and modality of the study were explained in advance to the mother before enrolment. Participation in the study was voluntary, and processing of mother and baby’s data was in accordance with the Privacy Act (General Data Protection Regulation—GDPR, EU Regulation 2016/679).

A retrospective observational study was then performed on the results of this survey by the Health Technology Assessment Committee of the teaching hospital, University of Catania, in collaboration with the Health Promotion Unit.

The original survey was performed on convenience samples, from several hospitals, of women willing to participate in the study after giving birth. All women in a ward at the moment of the presence of interviewers were invited, over a few days, except in case of multiple births, severe preterm child, or severe illness of the mother. As the survey had been performed in various types of hospitals, participating hospitals were labeled as follows. Community Hospitals: data from three small (less than 100 beds) public community hospitals, each providing about 500 deliveries per year; Emergency Hospital: a large public hospital, well known for emergency services, including public helicopter rescue service and an operating base for public ambulances, with over 1000 deliveries per year; Teaching Hospital: a teaching and research academic public hospital, providing childbirth services, with almost 2000 deliveries per year; Maternity Hospital: a specialized public hospital, providing only childbirth services, with roughly 1900 deliveries per year; Private Hospital: the largest of a few private health facilities in the area, accredited by the Regional Health Department, authorized for childbirths, with over 800 deliveries per year. These hospitals were compared with a “Project Hospital”: a specialized Mother and Child Health Department, providing over 2000 deliveries per year, located within a Public Hospital where a special project for breastfeeding advocacy allowed for social-psychological support by a psychology graduate to inform and support mothers immediately after delivery [20].

### 2.1. Questionnaire

The questionnaire presented 16 questions in two groups: general questions concerning the mother and the delivery; questions concerning the feeding of newborn, education, information provided to the mother before delivery, and rooming-in of the newborn.

Education level was classified as follows: primary = elementary and middle schools; secondary = high school; tertiary = university bachelor’s/master’s/doctoral degree. 

Feeding was classified as exclusive breastfeeding, if only breast milk was used and no other nutrients were provided; artificial, if the newborn had not been attached to the breast, and only baby formula or glucose gel had been provided; mixed, if both breastfeeding and artificial feeding had occurred.

Data were gathered in a Google Sheet document and transferred to a Microsoft Excel worksheet (Microsoft, Redmond, WA, USA) for subsequent analysis.

The percent coverage of this survey was evaluated by dividing the number of women enrolled during the enrolment time interval in each hospital by estimating the deliveries occurring in the same interval based on yearly deliveries obtained from the Department of Epidemiology of the Regional Government.

### 2.2. Statistical Analyses

Data were analyzed using Analyse-it for MS Excel 5.60 (Analyse-it Software Ltd., Leeds, UK). Results were reported as mean, standard deviation, and min–max range for numerical variables, and as percentages for categorical variables. Confidence intervals at the 95% level (95% CI) were also computed for selected results. Nonparametric tests were used for statistical testing when characteristics were categorical or ordinal, and the resulting probability level was reported whenever smaller than the set level *p* = 0.05. Kruskal–Wallis nonparametric test was employed followed by Steel–Dwass–Critchlow–Fligner multiple comparison test.

Logistic regression was applied for multivariate analysis of factors potentially influencing breastfeeding (Epi Info^TM^ v. 7.2.4.0, CDC, Atlanta, GA, USA). Logistic analysis requires that data are available for all variables of a case (questionnaire record), and imputation methods for missing data were not employed. 

The response was classified as 0 = exclusive breastfeeding and 1 = mixed or artificial feeding. In this way, according to Baglio [21], the odds ratios (OR) resulting from logistic regression convey the risk of abandoning/not up taking (OR > 1) or the protection from (OR < 1) abandoning/not up taking the exclusive breastfeeding. Available factors were expressed as follows: Age (years); Attending birth preparation courses (1 = yes); First childbirth (primiparae) (1 = yes); Delivery mode: 1 = Caesarean Section, 0 = Vaginal delivery; Newborn placement: 1 = Child in the room with the mother (Rooming-in), 0 = maternity ward. Breastfeeding started within 1 h from delivery: 1 = yes.

The mothers’ education level was expressed for analysis as two dummy variables: 0 = primary vs. 1 = secondary; and 0 = primary vs. 1 = tertiary.

For the purpose of logistic regression analysis, a set of dummy variables were created to compare results for each hospital type vs. the research project hospital. The resulting ORs express quantitatively how large a barrier in each type exists concerning exclusive breastfeeding results attainable with qualified assistance to the mothers.

## 3. Results

The survey analyzed the data collected through questionnaires administered at various times during 2016–2018 in the various birth hospitals. Overall, 3813 questionnaires were administered in different facilities providing maternity services in the province of Catania, Sicily, Italy, and 3368 questionnaires were complete and eligible for this study, after excluding records with sparse missing information. The number of questionnaires for each type of hospital was Project Hospital: 1180; Community Hosp.: 735; Teaching Hosp.: 531; Maternity Hosp. 432; Private Maternity Hosp. 396; Emergency Hosp.: 94. The coverage of the survey could be estimated as 38% with respect to the deliveries occurring in the time interval of administration of questionnaires.

Table 1 describes the characteristics of participating women, also reporting separately for exclusive breastfeeding and for mixed or artificial breastfeeding. Considering that mixed feeding is considered suboptimal [22], the percentage of mixed or artificial feeding was considered together as not carrying out exclusive breastfeeding at discharge.

Average age was not different in these two groups. In a preliminary logistic analysis, neither age in years nor age in range levels showed any significant contribution to explaining the breastfeeding response. Therefore, age was not considered further. This avoided list-wise deletion of records where age data were not available, allowing for all 3368 questionnaires to be studied with logistic regression. The average maternal age (31.4 ± 5.8 years) and the observed incidence of caesarean deliveries were generally similar to what was observed in this region of Italy by Italian Ministry of Health [23].

About half of the enrolled women were at their first delivery; rooming-in of the newborn in the same room with the mother was available in all facilities and adopted in most cases (90%). 

Table 1 also reports that only 32% of women had attended a prenatal course for birth preparation; therefore, 68% of women did not attend a prenatal course.

From birth to discharge, the percentage of women who breastfed was 96.5% (3250/3368); of these, 48% (1627) provided exclusive breastfeeding, whereas 48% (1623) had mixed breastfeeding, where in most occurrences (1391), baby formula was fed too; while in two, glucose gel (200 mg/kg) was used, and 22 were unspecified. For the remaining 118 babies, baby formula only was used, except for three cases, where glucose gel was given. Reasons reported for the inability to breastfeed were severe malformations, hospitalization in the Neonatal Intensive Care Unit (NICU), and maternal illness.

The possible contribution of the birth order to breastfeeding uptake was considered in a preliminary multivariate analysis of exclusive breastfeeding in women in their first, second, third, or further delivery. Results are summarized in Table 2, where statistical analysis failed to show significant differences, even for the slightly higher percentage of exclusive breastfeeding in primiparae; in the final logistic analysis, just first childbirth was compared to other parity levels pooled.

Similarly, the contribution of an earlier start of breastfeeding after delivery was considered in a preliminary multivariate analysis of exclusive breastfeeding in women who started within the 1st hour, in the 2nd–3rd hour or further, within 24 h or later. Results are summarized in Table 3, where statistical analysis showed a significantly higher percentage of exclusive breastfeeding when breastfeeding started within the first hour; in the final logistic analysis, this interval was compared to other timings pooled.

The percentages of women breastfeeding were different at various educational levels: among women with basic (primary) education the percentage of exclusive breastfeeding (EB) was 35% (314/900); among women who had attended high school (secondary) the percentage of EB was 50.5% (868/1718), whereas among women who attained a tertiary level (degree) the percentage of EB was 59% (445/750).

Table 4 reports the logistic analysis results of the questionnaire data, illustrating factors being significant barriers or facilitators to exclusive breastfeeding. As explained in methods, age did not play any significant role and was not considered as a factor in further analyses.

**Figure 1:**
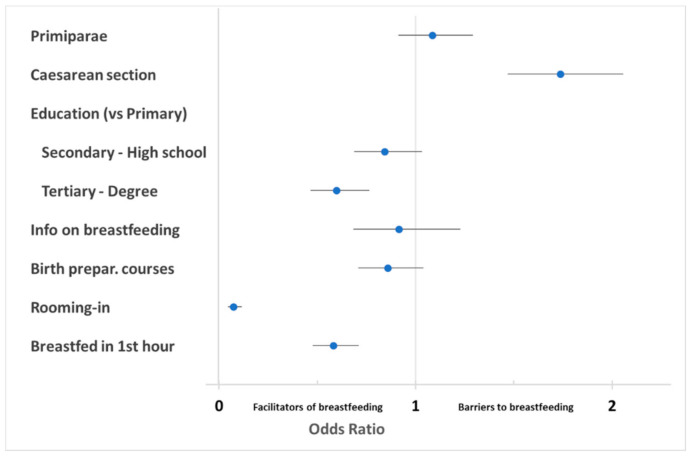
Odds ratios (OR) for maternal and obstetrical factors protecting from or increasing risk of abandoning/not up taking exclusive breastfeeding. Note: Forest plot of OR (with 95% confidence intervals) on a scale of relative risk, where 1 (vertical solid line) represents no difference, values below 1 indicate protection from abandoning breastfeeding, and values above 1 indicate increased risk of abandoning breastfeeding.

Figure 1 shows the protective factors and those that did not protect against the risk of abandoning breastfeeding. There are protective factors regarding the abandonment of breastfeeding: rooming-in; early onset of breastfeeding; a higher maternal level of education. Caesarean delivery increased the risk of abandonment.

**Figure 2:**
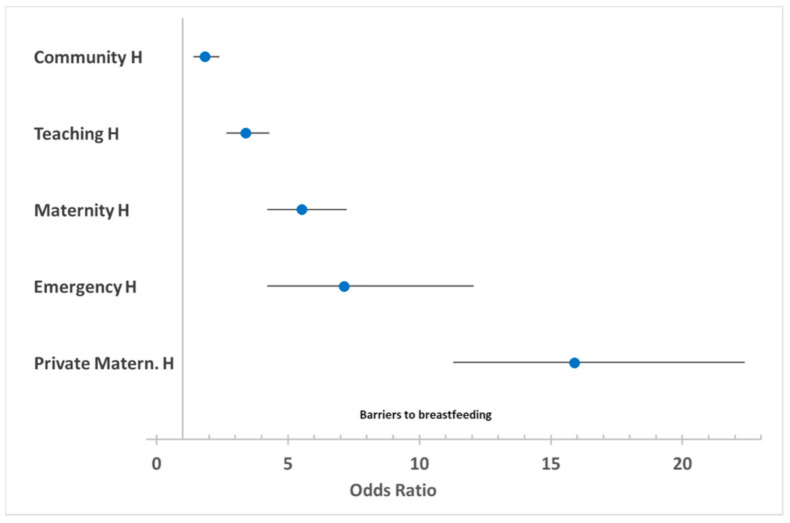
Odds ratios (OR) for various hospitals compared to a maternity ward where a breastfeeding advocacy program was introduced to help mothers start breastfeeding early after delivery and maintain exclusive breastfeeding instead of mixed or artificial feeding. Note: Forest plot of OR (with 95% confidence intervals) on a scale of relative risk, where 1 (vertical solid line) represents no difference, and values above 1 indicate increased risk of abandoning breastfeeding.

Figure 2 presents the OR for various types of hospitals compared to the maternity ward, where a breastfeeding advocacy program was introduced to help mothers start breastfeeding early after delivery and maintain exclusive breastfeeding instead of mixed or artificial feeding.

The data show that the risk of abandoning breastfeeding was significantly higher in all facilities providing maternity services compared to the special project, with the lowest risk in community hospitals and the highest risk of abandonment of breastfeeding in a private maternity hospital.

## 4. Discussion

From metropolitan areas in USA to rural African villages, it is known that a large majority of pregnant women declare a deep desire to breastfeed during pregnancy [24,25,26]. Despite the mother’s breastfeeding intentions, there is a gap between intentions and feeding practices. Various factors, including psychological, economic, and social factors, influence the mother’s desire to breastfeed [17,27,28,29,30,31].

To promote and encourage breastfeeding a multidisciplinary group (gynecologists, midwives, neonatologists, hygienists, psychologists, nurses, statisticians, quality managers, training and public relations managers,) was set up in our hospital. In order to offer useful advice, however, it was necessary to know the specific problems of the situation in which action is to be taken. The multidisciplinary group, coordinated by the Health Promotion Operating Unit, will use data presented in this work to focus on possible points for improvement breastfeeding within the obstetrics departments of our hospital. A hospital guideline will be prepared and distributed.

We focused our research on the relationship between the puerpera and the hospital providing maternity services in the Italian health system in a southern region. 

Among the mother’s characteristics, age was shown in preliminary analyses not to play a role influencing breastfeeding. A higher education level is confirmed as a facilitating breastfeeding factor [32,33]. As shown in Table 4, women with a higher educational qualification breastfed in a more significant proportion (59.6%; OR 0.60). Mothers who intend to breastfeed have more knowledge and confidence about nutrition and diet [34].

The order of childbirth did not show significant effects: the share of breastfeeding women did not significantly vary according to parity, and OR in primiparae was not significantly different from 1.

Among barriers, as already observed [35], having had a caesarean section moderately impaired breastfeeding uptake, with an odds ratio (OR = 1.74) indicating an almost double chance of declining exclusive breastfeeding. It is known [36,37] that women who have had a caesarean section breastfeed to a lesser extent, also because in these cases, favorable conditions to initiate breastfeeding (early breastfeeding and closeness of the baby to the mother during hospitalization) are more difficult to find.

Printed material or suggestions from friends, relatives, and doctors do not seem sufficient to change the new mother’s habits and ideas. Not even the information received from various sources (internet, TV, newspapers and magazines, friends or parents, etc.) by the mother seems to have a role in reducing the risk of abandonment of breastfeeding [34,35].

Employment issues are among the most significant barriers causing new mothers to stop breastfeeding early or neglect to start breastfeeding altogether [36]. 

Among the hospital’s facilitating factors, starting breastfeeding within 1 h from delivery showed a significant facilitating effect. Despite the WHO and the United Nations International Children’s Emergency Fund (UNICEF) policies advocating an early start, only 44% of infants initiate breastfeeding within the first hour after birth [38].

Our data confirm that rooming-in has a strong facilitating effect on exclusive breastfeeding [39,40,41,42]. Instead, and in contrast to previous reports [21,43,44], attendance to birth preparation courses did not appear to have a statistically significant positive effect. Given the variety of notions provided during these courses and the subjectivity of implementing what is explained, it is always difficult to evaluate their effectiveness [45].

These courses, which are proposed as a time of active promotion of the mother and the newborn’s health, do not seem able to overcome the objective difficulties encountered at the moment of birth in the hospital [46]. These courses indeed might increase maternal knowledge on relaxation and breathing techniques to be used during childbirth, eating habits during pregnancy, indications on neonatal care, and contraception in the puerperium.

Interestingly, among the types of hospitals considered, the risk of abandoning breastfeeding is lower in the one hospital where psychological support to new mothers was offered as a special ad hoc project. In this highly specialized hospital, the Mother & Child Health Department promoted a health services research project for breastfeeding advocacy, providing social–psychological support personnel to the mothers. The project was funded by the Regional Government within the Italian Healthcare Plan’s provisions, which provided funds dedicated to the protection of maternity and the birth path [20]. A special framework for innovative applied research in health services allowed social–psychological support by a psychology graduate to inform and support mothers immediately after delivery [19].

In the Maternity Hospital, where the breastfeeding support offered by a professional with psycho-pedagogical expertise was present, the percentage of exclusive breastfeeding was significantly higher than in all the other hospitals, where such a dedicated figure was not present. This unfavorable difference is moderate for the community hospitals, where a low number of births/days allow the social health staff to devote more attention to the new mothers. The difference is moderate also for the teaching hospital, where many professionals rotate close to the mother and the staff hired by the hospital (students of degree courses in Medicine and health professions, postgraduates, doctoral students, and fellows). 

The difference is high in maternity hospitals and emergency hospitals and very high in the private maternity hospital. In these facilities, the staff probably does not have the opportunity to devote time to accompany the mother in the first moments of breastfeeding, and there is no professional person dedicated to this function [31]. Health professionals are advised to create an atmosphere and an environment more suited to breastfeeding [47].

An active campaign favoring formula feeding could be at play in private settings, where formula milk sponsors could use relaxation and absence of pressure on the mother.

Support is a practice that reduces the abandonment of breastfeeding [48,49]. In the literature, the organization of home support is described as an attempt to regain as many women as possible to the practice of exclusive breastfeeding, at least for the first few months [50]. There are limited experiences that prenatal breastfeeding counselling, followed by sessions up to 4 months postpartum, can improve breastfeeding self-efficacy in mothers with previously unsuccessful breastfeeding [51].

The private hospitals historically represented in Italy the preferred place for giving birth, at times when public hospitals lacked comfort and accessibility, and are today much less used than 20 years ago. Evidence of some issues with safety for the mother and the child’s health has scaled down the volume of activity in private hospitals for delivery, and inadequacies of public hospitals have been overcome. 

In Italy, caesarean sections remain high both in public and even more in private facilities [52,53,54]. Over time, there has also been a push to reduce the fragmentation of having several private maternity hospitals with limited deliveries annually, and a concentration of public facilities too, where the occurrence of at least 500 births per year is required to keep the facility open. In 2016 it was reported that 89.2% of the births took place in public and equivalent healthcare institutions, 10.5% in private facilities, and just 0.1% elsewhere (other care facility and home). In total, 63.9% of deliveries took place in facilities, with at least 1000 deliveries occurring annually [23,55].

The reduction of the average length of stay in the hospital (2 days for vaginal delivery and max 5 days for caesarean) de-medicalized the birth event but probably reduced the time available to the mother and the baby to be accompanied in the first attempts at breastfeeding [36,56].

The issue of breastfeeding cannot be the prerogative of a single service: all the institutional actors around mother and child (from before conception, throughout the birth process, and the first year of life) play a decisive role [57,58,59].

The Baby Friendly Hospital Initiative (BFHI) and Baby Friendly Community Initiative promoted by the United Nations Children’s Fund (UNICEF) contain all the ingredients to promote breastfeeding with the active involvement of all components (mothers, health workers, policymakers, community actors), to promote information, education and organizational change in birth points. The beneficial effect of the BFHI in high-income countries is demonstrated [60], but the number of professional lactation counsellors must increase continuously to promote the full effect of the BFHI. 

Recognition of influencing factors could lead to better breastfeeding management in the days following hospital discharge [61,62].

Health professionals should create an atmosphere and an environment more suited to the promotion of breastfeeding [63]. In addition to evidence-based breastfeeding information, these professionals will have to provide practical guidance in a personalized way [64]. The health worker dedicated to following the mother immediately after giving birth will determine the success of the effective promotion more than all the prenatal courses and information previously received. A study reports that when the pediatric nurses took time and booked extra appointments, the mothers felt supported [65].

A limitation of this study is that, since the data of a previously administered questionnaire were collected, it was not possible from these to trace either the sources of information received (other than prenatal education) or information about family size, gestational age or nationality. It was not possible to have socioeconomic information regarding the mother’s income, employment or social resource/support that could interfere with the interpretation of the data that induce new mothers to stop breastfeeding early or not to start breastfeeding.

## 5. Conclusions

A lot of work is needed to improve care for infant feeding and early relationship building in all birth centers, public or private. This work must be carried out by a multidisciplinary group that studies the strengths and difficulties in each hospital regarding the promotion of breastfeeding. It is advisable to produce a written consensus, shared with all those who act at the time of delivery, to encourage breastfeeding and discourage the administration of artificial milk. Our study confirms how important it is that specialized health professionals act as “*breast feeding promoters*” that can facilitate early initiation and reduce the abandonment at the first difficulties. It is important, in all hospitals, to support the mother for breastfeeding in the first moments after childbirth to increase the number of those who will then continue with exclusive breastfeeding. This “*promoter”* could be especially helpful in encouraging and supporting women with low levels of education, caesarized women and where there is no rooming-in available.

More funding will be needed for breastfeeding programs to ensure the availability of qualified advice and the achievement of the WHO’s objectives [66].

The economic balance (the “bottom line”) for this effort would see any cost more than compensated by the savings in terms of burden of disease avoided for health consequences of forgoing breastfeeding.

## Figures and Tables

**Table 1:** Characteristics of women participating in the study, from questionnaires.

Factors Potentially Affecting Exclusive Breastfeeding (Risk of Cessation)	*n*		Exclusive Breastfeeding		Mixed or Artificial Breastfeeding	
No. of questionnaires	3368		1627		1741	
Age (yrs., mean ± std. dev.)	31.4 ± 5.8		31.1 ± 5.8		31.6 ± 5.7	
Age range (years, min-max)	15–58		15–52		15–58	
**(no. of available age data)**	(2920)		(1472)		(1448)	
Education level						
primary	900		314		586	
secondary	1718		868		850	
tertiary	750		445		305	
**No. of occurring characteristics** **in all questionnaires**		(%)		(%)		(%)
Primiparae	1647	(49)	832	(51)	815	(47)
Caesarean section	1319	(39)	503	(31)	816	(47)
Rooming-in	3046	(90)	1603	(99)	1443	(83)
Birth preparation courses	1082	(32)	592	(36)	490	(28)

**Table 2:** Uptake of exclusive breastfeeding in mothers according to the order of childbirth in their life (parity).

Order of Childbirth	*n*	%Exclusive Breastfeeding	(95% CI)
First child (primiparae)	1647	51	(48–53)
Second child	1217	47	(44–50)
Third child	386	44	(39–49)
Fourth child or more	118	46	(37–55)
Total	3368	48	(47–50)

No statistically significant differences revealed by Kruskal–Wallis test.

**Table 3:** Uptake of exclusive breastfeeding in mothers according to the delay in attaching the newborn to the breast, after delivery.

Start of Breastfeeding after Delivery	*n*	%Exclusive Breastfeeding		(95% CI)
**Within 1st hour**	2109	61	*	(59–63)
**Within 2–3 h**	380	34	§	(29–39)
**Further, within 24 h**	667	29	§	(26–33)
**Later on, before discharge**	108	8		(3–14)
**Never before discharge**	104	0		-
**Total**	3368	48		(47–50)

* Statistically different from all other groups; § Statistically different from groups 4 and 5; Kruskal–Wallis *p* < 0.0001, and multiple comparisons test.

**Table 4:** Odds ratios for predictive factors in logistic regression.

Factors Potentially Affecting Exclusive Breastfeeding (Risk of Cessation)	Odds Ratio	95% C.I.	*p*-Value
Education vs. primary level			
Secondary—high school	0.84	(0.69–1.03)	n.s.
Tertiary—degree	0.60	(0.47–0.77)	<0.00005
Primiparae (first childbirth)	1.09	(0.91–1.29)	n.s.
Caesarean section	1.74	(1.47–2.06)	<0.00005
Rooming-in	0.07	(0.05–0.12)	<0.00005
Breastfeeding started within 1 h	0.58	(0.48–0.71)	<0.00005
Birth preparation courses	0.86	(0.71–1.04)	n.s.
Other hospitals compared with Project Hospital §			
Community Hosp.	1.83	(1.41–2.39)	<0.00005
Teaching Hosp.	3.38	(2.67–4.29)	<0.00005
Maternity Hosp.	5.52	(4.21–7.23)	<0.00005
Emergency Hosp.	7.13	(4.21–12.06)	<0.00005
Private Maternity Hosp.	15.89	(11.28–22.38)	<0.00005

Odds ratios (OR) are listed with 95% confidence intervals and statistical significance: n.s. = not significantly different from 1 (no protective or barrier effect); *p*-value < 0.00005 = OR significantly different from 1, exerting a facilitating effect when OR < 1, a barrier effect when OR > 1. § Project Hospital = a highly specialized hospital, where the Mother&Child Health Department promoted a health services research project for breastfeeding advocacy, providing social–psychological support to the mothers.

## Data Availability

Data is available on request to the corresponding author (V.L.L.R.)
